# Whole genome expression profiling associates activation of unfolded protein response with impaired production and release of epinephrine after recurrent hypoglycemia

**DOI:** 10.1371/journal.pone.0172789

**Published:** 2017-02-24

**Authors:** Juhye Lena Kim, Edmund F. La Gamma, Todd Estabrook, Necla Kudrick, Bistra B. Nankova

**Affiliations:** 1 The Regional Neonatal Center, Maria Fareri Children’s Hospital at Westchester Medical Center, Valhalla, New York, United States of America; 2 Departments of Pediatrics, Biochemistry and Molecular Biology, Division of Newborn Medicine, New York Medical College, Valhalla, New York, United States of America; 3 New York Medical College School of Medicine, Valhalla, New York, United States of America; Duke University School of Medicine, UNITED STATES

## Abstract

Recurrent hypoglycemia can occur as a major complication of insulin replacement therapy, limiting the long-term health benefits of intense glycemic control in type 1 and advanced type 2 diabetic patients. It impairs the normal counter-regulatory hormonal and behavioral responses to glucose deprivation, a phenomenon known as hypoglycemia associated autonomic failure (HAAF). The molecular mechanisms leading to defective counter-regulation are not completely understood. We hypothesized that both neuronal (excessive cholinergic signaling between the splanchnic nerve fibers and the adrenal medulla) and humoral factors contribute to the impaired epinephrine production and release in HAAF. To gain further insight into the molecular mechanism(s) mediating the blunted epinephrine responses following recurrent hypoglycemia, we utilized a global gene expression profiling approach. We characterized the transcriptomes during recurrent (defective counter-regulation model) and acute hypoglycemia (normal counter-regulation group) in the adrenal medulla of normal Sprague-Dawley rats. Based on comparison analysis of differentially expressed genes, a set of unique genes that are activated only at specific time points after recurrent hypoglycemia were revealed. A complementary bioinformatics analysis of the functional category, pathway, and integrated network indicated activation of the unfolded protein response. Furthermore, at least three additional pathways/interaction networks altered in the adrenal medulla following recurrent hypoglycemia were identified, which may contribute to the impaired epinephrine secretion in HAAF: greatly increased neuropeptide signaling (proenkephalin, neuropeptide Y, galanin); altered ion homeostasis (Na+, K+, Ca^2+^) and downregulation of genes involved in Ca^2+^-dependent exocytosis of secretory vesicles. Given the pleiotropic effects of the unfolded protein response in different organs, involved in maintaining glucose homeostasis, these findings uncover broader general mechanisms that arise following recurrent hypoglycemia which may afford clinicians an opportunity to modulate the magnitude of HAAF syndrome.

## Introduction

Profound or recurrent hypoglycemia (RH) leads to increased morbidity and mortality with acute and chronic symptomatology and is a major public health problem in diabetic patients ([1], [2], [3]). Normally, counter-regulatory hormones (i.e. glucagon, epinephrine, cortisol, and growth hormone) oppose the actions of excessive insulin to reverse falling or dangerously low plasma glucose levels. Recurrent insulin-induced hypoglycemia blunts the counter-regulatory response (CRR) and the patient becomes unaware of the danger, a condition known as Hypoglycemia Associated Autonomic Failure or HAAF ([4], [5]). Interestingly, HAAF can be induced in healthy subjects, is observed in newborn infants with congenital hyperinsulinism, and can be demonstrated in animal models indicating that it represents a maladaptive physiologic response rather than a disease consequence of diabetes ([6], [7], [8], [9], [10], [11]).

The central nervous system mechanisms that contribute to HAAF have been extensively studied in humans and in animal models. Increasing evidence indicates their dominant role in the detection of hypoglycemia and initiation of CRR ([12], [13], [14], [15], [5], [16], [17], [18], [19], [20]). However, peripheral components of the sympathoadrenal system are also directly affected by RH ([21,22,23,24,25]). The molecular fundamentals underlying the defective counter-regulation remain elusive ([13]).

We have recently shown that the initial rise in tyrosine hydroxylase (TH; the rate limiting enzyme in the catecholamine biosynthesis) gene transcription, phosphorylation of the TH enzyme and induction of protein kinase A (PKA) activity in the adrenal medulla (AM) of normal Sprague-Dawley (SD) rats are similar whether measured during an acute single episode of hypoglycemia or during RH (an animal model of HAAF, [10]). This is consistent with normally functioning signaling between the CNS and the peripheral components of the sympathoadrenal system ([25]). However, the adrenal medulla’s cellular capacity to synthesize catecholamines was impaired in animals experiencing RH. This was evident by lower levels of adrenal TH mRNA attributed to enhanced degradation ([26], [27]) as well as accelerated inactivation of TH enzyme and lack of accumulation of TH protein at later time points following RH; all of which alone or collectively could affect the accumulation of epinephrine for release and indeed, correlated with attenuated epinephrine blood levels ([11], [25]). Given that the release and production of catecholamines in response to trans-synaptic stimulation is sustained for prolonged periods of time *in situ* ([28,29], [24]), but not during HAAF *in vivo*, additional regulatory factors or processes must exist in the intact animal to account for this ([30], [27], [26], [31]). To ascertain the widest possibilities of gene networks that may be involved in these mechanisms, we performed a whole genome expression analysis of adrenal medullary responses.

## Materials and methods

### Animals

Adult, male Sprague—Dawley rats weighing 285-320g with jugular vein (JV) and carotid artery (CA) catheter implants were purchased from Harlan Laboratories, Inc. Indianapolis, IN. Animals were individually housed in temperature (22°C) and humidity-controlled rooms and allowed access to food (regular rat chow, Agway Prolab 3,000; Syracuse, NY) and water ad libitum unless otherwise specified. The animals were acclimated to the animal facility and to handling for 3–4 days before experiments. Animal care and experimentation conformed to the Public Health Service Guide for Care and Use of Laboratory Animals and American Veterinary Medical Association Panel on Euthanasia Guidelines, and were approved by the Institutional Animal Care and Use Committee at New York Medical College.

#### Antecedent treatments

**Fig 1:**
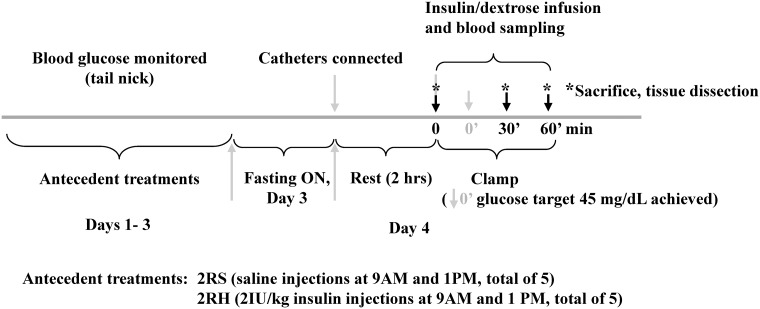
Experimental design. The schematic represents the basic protocol and the time line of the experiments. Before the antecedent treatments, a period of habituation was planned to minimize any external influences from travel and new housing. Body weight was monitored on a daily basis for each animal during the experiments.

The animals were randomly assigned to one of two experimental groups: sham-treated (recurrent saline injections twice daily at 9 am and 1 pm, 2RS) and insulin-treated (subjected to recurrent insulin-induced hypoglycemia twice daily, 2RH, see Fig 1). All treatments were for three consecutive days followed by a hyperinsulinemic-hypoglycemic glucose clamp on day 4. Antecedent hypoglycemic episodes were induced by intraperitoneal (i.p.) injection of regular human insulin (Humulin R, Eli Lilly, Indianapolis, IN) at a dose of 2 IU/kg body weight ([10], [11], [25]). Food was removed in all groups for 3 h after each insulin or saline injection. Blood glucose was monitored from tail nick samples using a handheld glucometer (AlphaTrak, Abbott Laboratories, Chicago, IL) every 30 minutes throughout each episode of hypoglycemia in 2RH group in order to achieve nadir glucose levels between 40 and 50 mg/dL (2.22–2.77 mmol/L).

**Fig 2:**
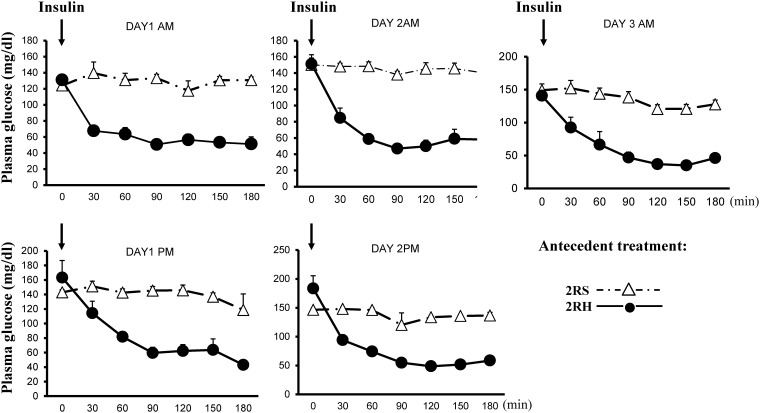
Blood glucose levels during the antecedent treatments on days 1–3. The values (mg/dL) are expressed as mean ± SE, n = 6.

Animals from the 2RS group were also subjected to the same treatments, except for the saline injections instead of insulin to avoid differences in any additional stress exposure (Fig 2). Hypoglycemia was terminated by providing the animals with solid food. Only one injection was given in the morning on the third day and the animals were fasted overnight before the glucose clamp studies began on the fourth day. The weight of each animal was monitored on a daily basis to ensure wellbeing and achievement of comparable nutrition.

#### Hyperinsulinemic-hypoglycemic clamp

**Fig 3:**
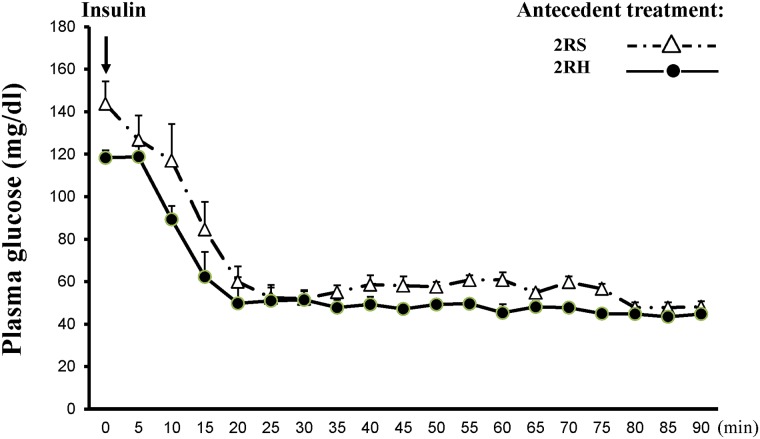
Plasma glucose concentrations during the hyperinsulinemic-hypoglycemic glucose clamp. The values (mg/dL) for twice-daily saline control (2RS) and twice daily RH groups (2RH) are shown as mean ± SE, n = 6 for each experimental group.

On the day of the experiment, the vascular catheters were connected to extension sets to minimize the stress from blood sampling via CA and dextrose/insulin infusion via JV ([25]). Animals were rested at least 2 hours before baseline sampling in order to let them recover from handling stress. A constant infusion of regular human insulin (50 mIU/kg/min; Eli Lilly) and a variable 20% dextrose infusion were started at 0’ and plasma glucose levels were monitored every 5 min (GM9 Analyzer, Analox Instruments Ltd, London, UK) to guide dextrose infusion. Plasma glucose levels were lowered to 45 mg/dL (2.5mmol/L) and maintained at this target until the end of the clamp study (Fig 3, [25], [11]). Blood samples were collected at 30 min intervals throughout the study, plasma was separated immediately and stored for subsequent hormonal analyses. Following each sample collection, the erythrocytes were resuspended in an equivalent volume of artificial plasma and infused back into the animal through the carotid artery to prevent volume depletion and anemia ([32], [11]). The animals (n = 6 per time point per group) were sacrificed either before (at 0 time point—baseline) or during (30’ and 60’ after achieving the target glucose levels) the clamp procedure with an overdose of sodium pentobarbital, followed by decapitation (Fig 1). The adrenal medullae were dissected and immediately frozen on dry ice. The tissues were stored at -80°C until further analyses.

### Hormone analyses

Plasma hormone concentrations (glucagon and insulin) were determined using commercially available radioimmunoassay kits from Linco Research, St. Charles, MO and Diagnostic Products, Los Angeles, CA ([33]). For measurement of catecholamine concentration, serum samples were analyzed using a competitive enzyme immunoassay (Rocky Mountain Diagnostics, Colorado Springs, CO) as described ([34], [11], [25]).

### Western blot analyses

Total protein extracts were prepared from each right adrenal medulla sample as described ([35]). Protein lysates were separated on 10% SDS- PAGE, electroblotted onto a nitrocellulose membrane (BioRad; Hercules, CA) and incubated with validated primary antibodies overnight (anti-Grp78 rabbit polyclonal antibody, dilution 1:1000 from GeneTex, cat. # GTX113340, Irvine CA; rabbit polyclonal anti-Derlin1- dilution 1:5000, ThermoFisher Scientific cat. # PA1-16598, Rockford, IL; rabbit polyclonal anti-TH—dilution 1:4000, from Novus Biologicals cat. # NB300-109, Littleton, CO). After incubation with secondary antibody (Goat Anti-Rabbit IgG, from Pierce, Rockford IL; diluted 1:30000) the immune reaction was visualized by enhanced chemiluminescent substrate from Pierce, utilizing a horseradish peroxidase label and Kodak XAR- 5 film, as described by the manufacturer. Blots were re-probed with primary antibody for housekeeping protein GAPDH (a rabbit polyclonal antibody from Sigma, St. Louis, MO, product number G9545) to confirm equal loading. The blots were exposed to autoradiography and the X-ray films were scanned and quantified with BioRad Quantity One software. For quantification, we always used a signal in the linear range. The ratios of immunoreactivity were calculated for each sample and the results are presented as fold induction compared to the corresponding control group on the same Western blot ([25]).

### Whole genome expression profiling

Total RNA was isolated from each left adrenal medulla sample by using RNA STAT-60 (Tel-Test, Inc, Friendswood, TX) and further purified as per Affymetrix^®^ guidelines. Pooled samples from each experimental group and each time point (n = 6 each) were subjected to microarray analyses using Rat Genome 230 2.0 array, Affymetrix (analyses performed at Yale Center for Genome Analysis, New Haven, CT). Microarray data was imported to Partek^®^ software for normalization by GCRMA methods, quantification of gene expression, and statistics. Genes showing altered expression with fold change >2 or <-2 were exported for functional annotation, pathway and comparison analysis by MetaCore^®^ (Thomson Reuter). Genes that are unique to each experiment group at different time point, and genes that overlap between any comparing groups were identified. The expression levels of selected genes were confirmed by real time RT-PCR analyses as described before ([25]). Raw and quantile-normalized microarray data and an associated project metadata file are available through the NCBI-GEO repository, access number GSE82145.

### Statistics

One-way analysis of variance (ANOVA) or repeated measures ANOVA, followed by a Neuman-Keuls post hoc analysis were used as appropriate. Statistics were performed using Sigma STAT/Plot software version 12 (Sigma, San Jose, CA). All data were expressed as means ± SE, P≤ 0.05.

## Results

### Plasma glucose and hormone levels

**Fig 4:**
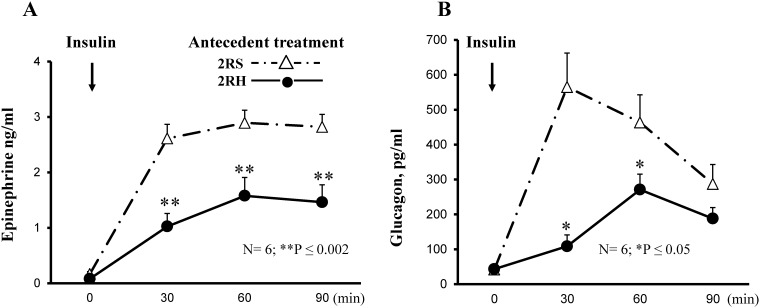
Plasma hormonal responses during the hypoglycemic clamp. A) Epinephrine (ng/ml) and B) glucagon (pg/ml) responses in twice daily recurrently hypoglycemic (2RH) rats and in the corresponding saline group (2RS). Data are summarized from three independent experiments, n = 6 animals per group. Values are shown as mean ± SE, *p <0.05 or **p<0.002 vs. corresponding control at given time point.

The experimental design of the current study is illustrated on Fig 1. RH was produced by twice daily i.p. injections of insulin (2IU/kg) for 3 days as we described before ([11], [25]). The target blood glucose levels during the antecedent insulin treatments were 45–50 mg/dL in the 2RH experimental group (see time course of blood glucose decline on Fig 2). There were no significant differences in blood glucose levels between individual animals (n = 6) in the 2RH group at any time point tested. Control animals received saline (2RS group). After overnight fast all animals (from both, 2RH and 2RS groups) were subjected to hyperinsulinemic-hypoglycemic clamp on day 4. A set of animals from both experimental groups were sacrificed at baseline (before insulin infusion, 0 time point). Once target plasma glucose levels between 40–45 mg/dL for each individual animal were achieved (0’ time point), a set of animals from each study group were sacrificed 30 (time point 30’) and 60 minutes later (time point 60’ on Fig 1) and tissues were dissected for analyses as described in the methods section of the manuscript. There were no significant differences in the plasma glucose levels between individual animals from each group and between the groups at each time point studied during the clamp (Fig 3). Also, plasma insulin concentrations were measured and found not significantly different between 2RH and 2RS groups at baseline and at the end of the clamp (data not shown), ensuring that the rats were exposed to the same glucose and insulin stimuli and only differed in the antecedent history between the comparison groups. Baseline serum concentrations of epinephrine and glucagon were also measured prior to the initiation of the clamp on day 4 (Fig 4A and 4B) and no significant difference was found between both experimental groups. Both epinephrine and glucagon levels rose significantly as insulin infusion began in both groups. However, the magnitude of the rise was significantly attenuated in 2RH group as expected, thus confirming proper implementation of the HAAF model. Corticosterone responses were also evaluated, which increased significantly from the baseline values during the clamp in both groups and did not display significant differences between the groups (results not shown).

### Differential gene expression profiles triggered by acute hypoglycemia and RH

To identify potential cellular targets during acute hypoglycemia and RH, we used microarray technology allowing genome-wide simultaneous measuring of changes in gene expression. Total RNA was isolated from individual AM samples, pooled from 6 animals for each specific time point/experimental group and subjected to microarray analysis as described in methods. Differentially expressed genes (DEGs) were identified and ranked according to significant up-regulation or down-regulation of two fold or more (*P≤ 0.05). Fold-change criteria combined with a minimum *P*-value cut off derived from an appropriate *t*-test is considered a straightforward method for identifying DEGs ([36]).

At baseline (0 time point—before insulin infusion on day 4, see Fig 1) a total of 167 genes were significantly altered in the 2RH group (vs. 2RS group): from them, 72 were up-regulated and 95 were down-regulated (top 10 genes for each category listed on Table 1, for complete list of DEGs see S1 Table). During the hyperinsulinemic-hypoglycemic clamp, the number of genes affected by RH increased. 30 min after achieving the target plasma glucose levels total of 728 transcripts were significantly altered, 402 up-regulated and 326 down-regulated (2RH30 vs. 2RH0). At 60 min these numbers were 933, 515 and 418 respectively; Table 2, S2 Table. In animals exposed to acute hypoglycemia (2RS group) a total of 1,122 transcripts were found significantly altered at the 60’ min time point, 620 up-regulated and 502 down-regulated (2RS60 vs 2RS0).

**Table 1:** DEGs with highest fold change by RH at baseline (2RH0 vs. 2RS0).

Gene Title	Gene Symbol	RefSeqTranscript ID	Fold change
Neurotensin	Nts	NM_001102381	↑106.364
Proenkephalin	Penk	NM_017139	↑29.329
Galanin/GMAP prepropeptide	Gal	NM_033237	↑15.726
Neuromedin U	Nmu	NM_022239	↑11.486
Lipocalin 2	Lcn2	NM_130741	↑9.038
TIMP metallopeptidase inhibitor1	Timp1	NM_053819	↑8.067
VGF nerve growth factor inducible	Vgf	NM_030997	↑7.745
Prolactin releasing hormone	Prlh	NM_022222	↑6.515
Pro-neuropeptide Y-like	NPY	NM_012614	↑5.201
Myosin heavy chain B	MyhB	NM_001100485	↑5.089
Neurotrophic tyrosine kinase receptor type 1	Ntrk1	NM_021589	↓4.15
Cleavage and polyadenylation specific factor 4	Cpsf4	NM_001012351	↓3.528
Bradykinin receptor B2	Bdkrb2	NM_001270713	↓3.465
Kelch-like family member 8	Klhl8	NM_001105995	↓3.378
Centrosomal protein 95kDa	Cep95	NM_001013862	↓3.378
Sodium channel, voltage gated type III beta	Scn3	NM_139097	↓3.359
Diphthamide biosynthesis 1	Dph1	NM_001105809	↓3.088
Ubiquitin specific peptidase	Usp3	NM_001025424	↓3.077
cAMP response element modulator	Crem	NM_001110860	↓3.017
Hyaluronan and proteoglycan link protein 4	Hapln4	NM_001108398	↓2.977

**Table 2:** DEGs with highest fold change by RH at 60 min (2RH60 vs. 2RS60).

Gene Title	Gene Symbol	RefSeqTranscript ID	Fold change
Gamma-2a immunoglobulin heavy chain	IgG-2a	XM_002727307	↑11.336
Protein phosphatase 2, regulatory subunit B, alpha	Ppp2r2a	NM_053999	↑6.732
Chemokine (C-X-C motif) ligand 10	Cxcl10	NM_139089	↑5.259
RT1 class Ia, locus A2	RT1-A2	NM_001008829	↑3.779
Interferon regulatory factor 7	Irf7	NM_001033691	↑3.717
Lipocalin 2	Lcn2	NM_130741	↑3.431
Anillin, actin binding protein-like 1	Anln1	XM_006222448	↑3.197
Cytochrome P450 family 2C popypeptide 7	Cyp2c7	NM_017158	↑2.897
Suppressor of cytokine signaling 3	Socs3	NM_053565	↑2.874
Transcription elongation factor B (SIII), polypeptide 1	Tceb1	NM_001270561	↑2.696
Suppression of tumorgenicity 18	St18	NM_153310	↓6.019
AT rich interactive domain 1B	Arid1b	NM_172157	↓5.436
Paternally expressed 10	Peg10	XM_006224893	↓4.761
Yippee-like 4 (Drosophila)	Ypel4	NM_001024369	↓3.930
Regulator of G-protein signaling 4	Rgs4	NM_017214	↓3.722
Neuregulin 1	Nrg1	NM_001271118	↓3.592
K^+^voltage-gated channel subfamily G	Kcng3	NM_001033957	↓3.367
G protein-coupled receptor 19	Gpr19	NM_080579	↓3.330
Calcyon neuro-specific vesicular protein	Caly	NM_001190399	↓3.239
Protein phosphatase 2, regulatory subunit B’ epsilon isoform	Ppp2r5e	NM_001106740	↓3.212

**Fig 5:**
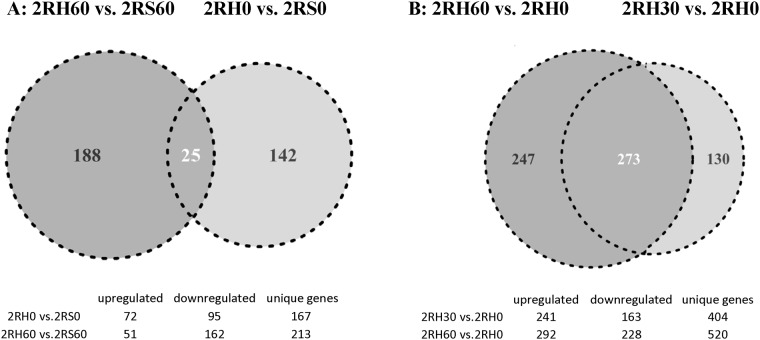
Venn diagrams of differentially expressed genes. A) Comparative analysis of DEGs in the 2RH0 and 2RH60 groups vs their respective saline controls (2RS0 and 2RS60); B) Comparative analysis of DEGs in 2RH groups—2RH30 and 2RH60 vs 2RHO respectively.

We also performed a comparative analysis between the differentially expressed genes in adrenal medulla during acute hypoglycemia and RH. While a variety of comparisons can be drawn from the data set, our aim was to detect changes unique to RH at different time points. The results from two of the comparisons are illustrated on Fig 5. A relatively small number of DEGs was identified to be overlapping (common) between the animals exposed to acute hypoglycemia (2RS) and RH (2RH) (25, listed on Table 3, S1 Table). Several genes were up-regulated at both baseline (before insulin infusion on day 4) and 60 min after achieving the target glucose levels during the hyperinsulinemic-hypoglycemic clamp. A total of 16 were down-regulated and the expression of one gene (Get4, Goldgi to ER traffic protein 4) was induced at the 0 time point and inhibited at the 60 min time point (shown in bold on the Table 3). A total of 167 genes were identified as unique for the 2RH0 group (animals with previous history of RH) at baseline, both induced (72) and suppressed (95) compared to the 2RS0 experimental group. At the 60 min time point the number of unique genes in the 2RH60 group (vs. maximal response group 2RS60) increased to a total of 213 (51 up-regulated and 162 down-regulated, respectively), see S1 Table.

**Table 3:** List of common DEGs during acute hypoglycemia and RH.

Gene Title	Gene Symbol	2RH0/2RS0	2RH60/ 2RS60
anillin	Anln	↑4.36	↑3.19
Chemokine (CXC) ligand 10	Cxcl10	↑3.02	↑5.25
gamma-2a immunoglobulin heavy chain	IgG-2a	↑4.69	↑11.33
lipocalin 2	Lcn2	↑9.038	↑3.43
RT1 class 1a, locus A2	RT1-A2	↑2.62	↑3.78
transcription elongation factor B, polypeptide 1	Tceb1	↑2.31	↑2.69
bradykinin receptor B2	Bdkrb2	↓3.46	↓2.29
Calcyon neuron-specific vesicular protein	Caly	↓2.66	↓3.24
caspase 6	Casp6	↓2.36	↓2.25
diphthamide biosynthesis 1	Dph1	↓3.09	↓2.83
ELAV like RNA binding protein 1	ElavL1	↓2.03	↓2.13
glutamyl-prolyl-tRNA synthase	Eprs	↓2.37	↓2.11
**Goldgi to ER traffic protein 4**	**Get4**	↑**2.1**	↓**2.46**
Guanine nucleotide binding protein α activating polypeptide 0	Gnao1	↓2.48	↓2.53
hyaluronan and proteoglycan link protein 4	Hapln4	↓2.98	↓2.17
neurotrophic tyrosine kinase receptor type 1	Ntrk1	↓4.15	↓2.17
parkinson protein 2, E3 ubiquitin ligase	Park2	↓2.42	↓2.84
regulator of G-protein signaling 4	Rgs4	↓2.29	↓3.72
SAP domain containing ribonucleoprotein	Sarnp	↓2.44	↓2.3
solute carrier family (Na/K/Ca exchanger), 2	Slc24	↓2.02	↓2.13
solute carrier family 6 (neurotransmitter transporter), 4	Slc6a4	↓2.03	↓2.17
tetraspanin	Tspan18	↓2.04	↓2.02

The comparison between RH samples taken at different time points before and during the clamp revealed a total of a 404 genes affected at 30 min (241 induced and 163 down-regulated) and these numbers further increased to 520 at 60 min (Fig 5B). From them, 273 genes were common (overlapping) for all conditions, and 130 genes were unique for 2RH30 experimental group and 247 for the 2RH60 group, suggesting dynamic changes in the adrenal transcriptome during the hyperinsulinemic clamp.

### Enrichment analysis of differentially expressed genes

**Fig 6:**
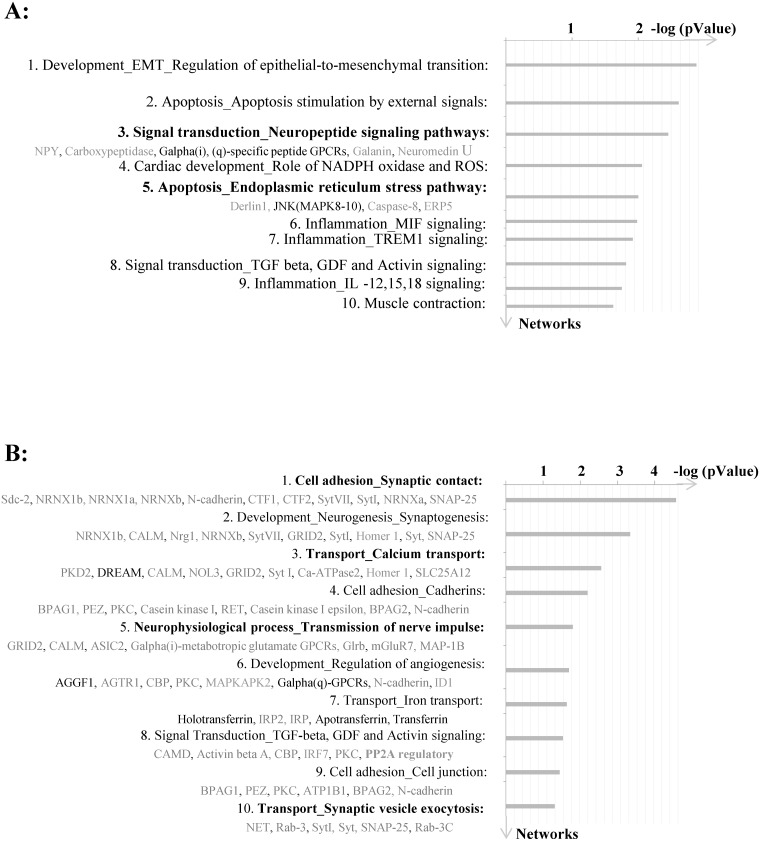
Enrichment analysis of DEGs in 2RH and 2RS experimental groups. A) Shown is the distribution by process networks at 0 time point (2RH0 vs. 2RS0) and B) at 60 min time point (2RH60 vs. 2RS60). Sorting is done for unique genes and both signals (induced genes—shown in red and repressed genes—in blue) included. Top 10 process networks are listed based on their—log (p-value). For list of abbreviations see supplemental file S1 Text.

To define which biological processes are switched on or off during RH, we performed gene enrichment analysis in four different functional ontologies: canonical pathway maps, process networks, disease categories and gene ontologies (GO) using MetaCore^™^. This allowed us to analyze functionally related genes (for example, genes belonging to a specific biochemical process) as a whole. The results for unique DEGs in the RH group are shown on Fig 6. Among the top 10 process networks (2RH0 vs.2RS0) were neuropeptide signaling and endoplasmic reticulum (ER) stress pathways (Fig 6A). RH predominantly affected genes involved in cell adhesion, synaptic contact, calcium transport, transmission of nerve impulse and synaptic vesicle exocytosis. Most of them were significantly down-regulated compared to the maximal response group 2RS60 (animals exposed acute hypoglycemia, see distribution by process networks for unique genes shown on Fig 6B).

**Fig 7:**
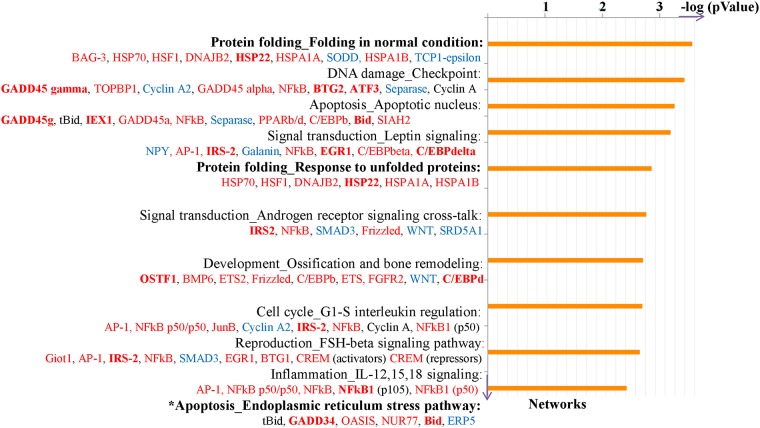
Functional analysis of DEGs at different time points following RH. The distribution by process networks is shown, with top 10 significantly enriched GO items for differentially-expressed common genes in 2RH30 vs 2RH0 and 2RH60 vs 2RH0. For list of abbreviation see S3.

**Fig 8:**
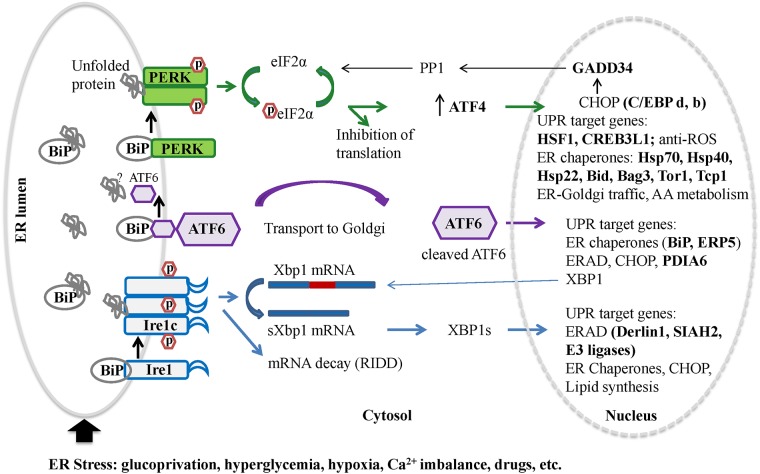
RH induces the unfolded protein response in rat adrenal medulla. A schematic of the unfolded protein response in mammals is presented. In resting cells all three ER stress sensors are inactive due to association with Grp78. Accumulation of unfolded proteins leads to dissociation of Grp78 and activation of IRE1, ATF6 and PERK which reprogram transcription and translation in a concerted manner to restore homeostasis: increase the transcription of genes involved in protein folding (ATF6 and IRE1 signaling), attenuate global protein synthesis (PERK) by phosphorylating translation initiation factor 2 (eIF2a) while promoting the translation of ATF4. ATF4 controls the expression of CHOP, which in turn induces GADD34 –a negative feedback loop effector that terminates UPR signaling by recruiting protein phosphatase1 catalytic subunit resulting in dephosphorylation of eIF2a and recovery of protein synthesis ([37]). Selected genes affected only by RH in our experiments are indicated in bold.

We also followed the dynamic changes in adrenal gene expression during the hyperinsulinemic-hypoglycemic clamp. The results for 2RH group are illustrated on Fig 7 (distribution by process networks for overlapping genes in the 2RH group, comparison on Fig 5B). Several key process categories were affected by RH including protein folding in normal condition, apoptosis/ apoptotic nucleus and signaling/leptin signaling. Interestingly, number of genes associated with the unfolded protein response (UPR) were induced at different time points of RH. Among these were mRNAs encoding Derlin-1, Cebpb, (member of the CHOP family of proteins) as well as several chaperons (such as members of the heat shock protein family- Hsp22, Hsp70, Hsp40; as well as Tor1A and Tcp1). Fig 8 summarizes UPR pathways with the genes detected with altered expression in our analysis (shown in bold).

### RH induces the expression of Grp78—Master initiator of UPR

**Fig 9:**
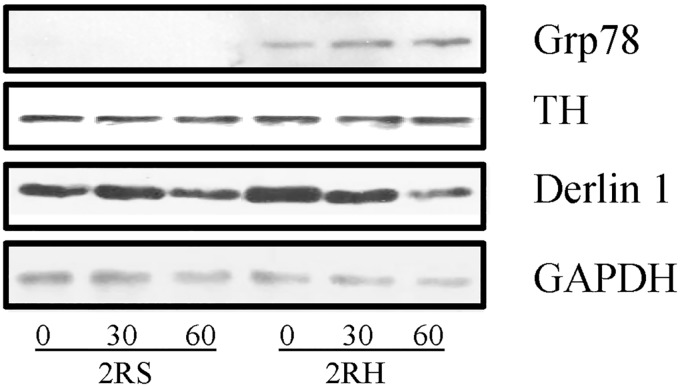
Western blot analysis confirms activation of UPR following RH. Total protein lysates from the right adrenal medulla of saline (2RS) and RH groups (2RH) were subjected to Western blot analyses as described in methods. Proteins were separated by SDS-PAGE, electroblotted and the membranes were sequentially probed with antibodies specific to Grp78, Derlin1, TH and GAPDH. Similar results were obtained in two separate replicate experiments.

A significantly increased amounts of glucose regulated protein (GRP) 78 protein over baseline expression has become an established indicator and marker for the presence of cellular ER stress (rev. in [38]). To confirm the induction of UPR in RH we performed western blot analysis as described in methods section (Fig 9). Grp78 was not detectable at any time point during acute hypoglycemia (2RS group). However, in the 2RH group, the presence of GRP78 was evident at baseline and increased >2–3 fold during the clamp (at 30 and 60 min), consistent with the induction of the UPR. In addition, Derlin-1 protein, functional component of the ER-associated degradation (ERAD) pathway for misfolded luminal proteins [39], was detectable in all protein lysates on the same blots. Notably, the relative Derlin-1 immunoreactivity was significantly increased in 2RH0 group, followed by decline to baseline levels (in 2RH60), consistent with the microarray data.

## Discussion

In the current study we discovered a novel association between activation of UPR signaling following recurrent glucoprivation and the defective counter-regulatory response that was evident even in otherwise normal rats. For the first time our whole genome expression profiling approach illustrated DEGs in the adrenal medulla of rats that were unique to the exposure to RH. We showed that among the RH-activated networks are ER stress and UPR, including up-regulation of UPR-related chaperones (Grp78 master initiator of UPR and other members of the Hsp70 family; Dnajb2, Hsp22, Tcp and Tor1). We also identified up-regulation of transcription factors (ATF4, Cebpb, Cebpd, CREB3L1, CREB3L3, and HSF1) and proteins involved in ERAD (Derlin-1, ERP5, SIAH, E3 ligases; Figs 7 and 8). This is the first report of the potential role of UPR in TH biology or during HAAF. We speculate that the induction of UPR-related general inhibition of translation and potentiated decay of ER-localized mRNAs together with the disturbed Ca^2+^ homeostasis and suppression of the regulated secretory pathway appear to contribute significantly to the altered regulation and diminution of adrenal catecholamine production and release in HAAF.

### A prior exposure to RH results in prolonged changes in adrenal gene expression profiles

Through gene expression profiling, a total of 1,122 differentially-expressed genes were identified in response to acute hypoglycemia in the current study (genes altered two-fold or more at 60 min time point vs 0 time point on day 4 –Fig 1, 2RS60 vs 2RS0). This number was similar (1,100 DEGs) for the animals exposed to RH (2RH60 vs 2RS0) confirming that in both cases there was a significant transcriptional re-programming of adrenal chromaffin cells. Notably, several genes remained affected even 20 hrs after the last antecedent episode of RH (2RH0 vs 2RS0, Figs 5A and 6A, for complete list of unique genes see S1 Table). Among the top score process networks identified for unique genes in the 2RH group at baseline were apoptosis, neuropeptide signaling and interestingly, ER stress (Fig 6A).

The mRNAs encoding both, Derlin-1 and ERP5 (also named Protein disulfide isomerase (EC 5.3.4.1), PDIA6) were significantly upregulated in the 2RH0 group, compared to the 2RS0 (Fig 6A and S1 Table). The ER not only provides mechanisms to facilitate folding of newly synthesized secretory and membrane proteins, but also harbors molecular machineries that eliminate proteins that fail to fold or assemble correctly ([40]). We confirmed the significant upregulation of Derlin-1 protein during RH by Western blot (Fig 9). While altered expression of Derlin-1 was not associated with HAAF so far, physiological or pathological changes in Derlin-1 expression levels have been shown to affect glucose-stimulated insulin secretion by altering the surface expression of ATP-sensitive potassium channels ([39]).

ERP5 is known to catalyze formation, reduction, and isomerization of disulfide bonds in proteins, and to play a role in folding of disulfide-bonded proteins ([41]). It reacts with substrates that are known to associate with Grp78, including those targeted for ER-associated degradation ([42]). In this regard, we also identified an increase in Grp78 immunoreactivity in the 2RH group, evident at baseline and gradually enhanced during the clamp portion of the experiment (Fig 9), thus confirming the induction of ER stress in the AM of animals exposed to RH.

In many cell types glucose uptake occurs by facilitated diffusion and is affected by blood glucose concentration ([43]). Our results support the notion that in addition to the well described CNS-mediated trans-synaptic effects of hypoglycemia, deranged glucose fluxes in central and peripheral tissues, including AM caused by antecedent glucoprivation, may disturb cell homeostasis inducing ER stress and UPR.

Another set of genes, encoding several neuropeptides (including PENK, NPY and galanin) which are co-released with catecholamines in neuronal activity-dependent manner ([44]), were also significantly increased in the 2RH0 group (Table 1, Fig 6A, S1 Table). Our data are consistent with previously reported long lasting overexpression of several neuropeptides in response to stress (including acute hypoglycemia, [45], [46], [47]). These elevated neuropeptides can be logically assigned relevant to the defective CRR in HAAF: opioids exert an almost universal suppressive paracrine effect on the secretion of classical neurotransmitters, neuropeptides, and hormones in neurons, adrenal chromaffin and other cells ([48]). Furthermore, opioid receptor blockage has been shown to prevent HAAF ([49,50]). NPY is also known to exert a negative feedback loop on TH expression and both, NPY ([51], [52], [53]) and galanin [54], [55] can control adrenal secretory capacity. Recently NPY expression was found required for fasting-induced autonomic synaptic plasticity at the preganglionic-chromaffin cell synapse ([56]).

### Unique genes and process networks/pathways induced during the hyperinsulinemic—hypoglycemic clamp in animals, previously exposed to RH

In the current study a total of 213 adrenal genes were identified as unique for the 2RH60 group (vs. 2RS60 –maximal response to hypoglycemia group). From them, 51 were significantly induced and 162 were suppressed (Fig 5A). The majority of the down regulated genes belonged to process networks involved in synaptic contact/cell adhesion (Scd2, Syt, NRNX, SNAP25), calcium transport (PKD2, CALM, NOL3, Ca^2+^–ATPase 2), transmission of nerve impulse, and exocytosis (NET, Rab3, Syt, SNAP25) (Fig 6B). These include genes encoding synaptotagmins—synaptic vesicle membrane proteins abundant in nerve and some endocrine cells proposed to function as calcium sensors in the regulation of neurotransmitter release and hormone secretion ([57, 58]). More specifically, Syt1 is recognized as a Ca^2+^ sensor for fast synchronous neurotransmitter release in forebrain neurons and chromaffin cells ([59, 60]) and Syt7 as a major Ca^2+^ sensor for exocytosis in chromaffin cells [61,62]. Recently it was found that Syt1 and Syt7 play an essential overlapping role in maintaining the readily- releasable pool of vesicles, in addition to their gene-specific function as Ca^2+^ sensors and fusion clamps [61].

Ca-binding synaptotagmins are involved in both, early synaptic vesicle docking to the presynaptic membrane (via interaction with neurexin beta or SNAP25) and the late steps of synaptic vesicle fusion with the presynaptic membrane ([63]). Those genes were also found down regulated in our study. Our results suggest that the observed down regulation of multiple genes involved in Ca^2+^ transport, synaptic contact and exocytosis and the developing of ER stress/activation of the UPR response in adrenal medulla of repeatedly hypoglycemic animals may be related to the reduced epinephrine secretion during HAAF.

It is worth mentioning that the gene encoding protein phosphatase 2 regulatory subunit B (PP2A) is among the few unique genes significantly induced in the 2RH60 group in our study (Fig 6B, S1 Table). PP2A is one of the four major Ser/Thr phosphatases with diverse function in the cell (rev. in [64]) and its B subunit might modulate substrate selectivity and catalytic activity. Interestingly the increased expression of PP2A (known to dephosphorylate TH enzyme *in vitro*—[65], [66]) correlated with the decline in phosphorylated TH enzyme at later time points in the 2RH group ([25]). Thus we speculate that ER stress and activation of UPR may account for the posttranscriptional regulation of TH previously reported by us during HAAF where both, TH mRNA longevity and TH translation are reduced (due to IRE1-dependent decay of ER membrane-associated mRNAs [67] and global inhibition of translation induced by UPR activation [68]). They would also promote faster inactivation of TH enzyme (either via PP2A mediated dephosphorylation at Ser40 or proteasomal degradation by the ERAD [69]). In this regard, one earlier study has demonstrated that a fraction of TH enzyme is an integral component of bovine chromaffin granule membranes ([70]) and could thus serve a purpose in coordinating TH activity and catecholamine release.

A potential contribution of adrenal ubiquitin proteasome system and ER stress in blunting the sympathoadrenal responses in HAAF has not been reported before. Accumulating evidence indicates that ER stress-mediated cell dysfunction and death is involved in the pathogenesis of human chronic disorders including metabolic diseases (obesity and Type 2-diabetes) and neurodegeneration ([38]), and UPR has been a growing subject of extensive investigations as a potential therapeutic target ([71], [72], [73], [74], [75], [76], [77], [78], [79], [80], [81]). Although the possible impact of chronic adrenal ER stress in the development of HAAF has not been tested previously, it has been shown that acute hypoglycemia in rodents increases several biochemical markers of the UPR and glucose production in the liver (not in the kidneys or the pancreas [82]).

### Evidence for activation of adrenal UPR during subsequent episode of hypoglycemia in RH animals

Pathway analysis indicated that “*Protein folding*” was the most significantly enriched item in the distribution by process network for common genes (2RH30 vs.2RH0 and 2RH60 vs. 2RH0, Fig 7, comparison shown on Fig 5B) and “Response to unfolded proteins” was in the top 5 scored networks. A gene set for “Protein folding and response to unfolded proteins” includes molecular chaperons, members of the 70 kDa heat shock protein family (HSP70, HSPA1A, HSPA1B), 40kDa heat shock protein family (DNAJB2), small heat shock proteins (HSP22), heat shock factor 1 (HSF1) and BCL2-associated athanogene 3 (BAG3 –co-chaperone shown to regulate formation of SNARE complex and insulin secretion in beta cells [83]), indicating that activation of UPR may be a significant aspect of the altered responses to hypoglycemia and defective CRR in HAAF.

Diverse physiological or pathological challenges can provoke ER stress and activate the set of intracellular signaling pathways termed the UPR ([75]). Beneficial outputs of UPR restore homeostasis and normal ER functions, while destructive outputs trigger programmed cell death ([84], [85], [38]). Prior to this report, activation of UPR in the adrenal medulla has not been described in HAAF, yet it has been implicated in the proper functioning and survival of pancreatic islet beta cells in Type 1 and Type 2 diabetes ([77], [86]). Accumulation of unfolded or misfolded proteins in the ER causes the dissociation of Grp78 chaperone from the three widely expressed ER transmembrane sensors: protein kinase RNA (PKR)-like ER kinase (PERK), activating transcription factor-6 (ATF6), and inositol-requiring enzyme-1 (IRE1a), eliciting the UPR. Combinatorial signals from IRE1a, PERK, and ATF6 initially trigger transcriptional programs that up-regulate genes encoding many ER chaperones, oxidoreductases, and ERAD components ([38]). The UPR also imposes a transient translational block during ER stress and promotes decay of ER-localized mRNAs ([87]) in a stress-dependent manner, thereby concentrating available resources to allow preexisting proteins to fold before new ones are made (rev. in [88], see Fig 8). Stress levels in the ER are reflected in the degree of activation of IRE1a, PERK, and ATF6; therefore, these upstream sensors are centrally poised to receive and transmit the information needed by the cells to commit in a dichotomous manner either toward adaptation or self-destruction.

#### Limitations of design

Our study was not designed to address causality in our integrative view of the altered stress responses following RH. The cause and effect relationships between ER stress, UPR and the attenuated epinephrine responses in HAAF will be tested in validation experiments in the future. In addition, in our microarray analysis data we selected relatively high p-value cut off (two-fold and more) and the actual number of genes with biologically significant changes in gene expression could be higher. It should be also mentioned that our observation and the interpretation of the results are based on few time points studied—before and during the exposure to acute hypoglycemia and RH. Given the highly dynamic changes in variety of homeostatic processes we may have missed some events. Moreover, we measured the mRNA levels, not the protein or its function.

## Conclusions

We used microarray experiments to uncover genes regulated by acute hypoglycemia and RH in the adrenal medulla of normal SD rats. Overall, the reported observation underscores a physiologically important role for ER stress and the UPR to alter the peripheral sympathoadrenal components of the stress response to hypoglycemia. To date, no mechanisms linking the attenuated adrenal epinephrine response in HAAF to ER stress and UPR have been described. Although we do not provide direct evidence to support this hypothesis, we propose that the epinephrine responses to hypoglycemia result from a complex and dynamic interplay between centrally mediated trans-synaptic stimulation of catecholamine synthesis and release, as well as the opposing effects of cellular ER stress and the activation of UPR caused by glucoprivation. Further functional studies are necessary to confirm these correlations and validate the biological significance potentially leading to the useful therapeutic targets for many metabolic disorders.

## Supporting information

S1 Table Comparison analysis of DEGs during acute hypoglycemia and RH. DEGs were identified for 0 time point (before) and 60 min time point (during the hypoglycemic clamp) for 2RS and 2RH experimental groups. Overlapping genes regulated at all conditions and genes unique for each time point (2RH0 compared to 2RS0 and 2RH60 compared to 2RS60) are listed on separate spreadsheets.
(XLSX)

S2 Table Comparison analysis of DEGs at different time points during RH. DEGs were identified before (at 0 time point) and during (at 30 and 60 min) the hypoglycemic clamp in the 2RH experimental group. DEGs common for all conditions, and genes unique for 2RH30 (vs. 2RH0) and for 2RH60 (vs. 2RH0) are listed on separate spreadsheets.
(XLSX)

S1 Text Abbreviations for Figs 6 and 7. (DOCX)
