# Correction: Whole genome expression profiling associates activation of unfolded protein response with impaired production and release of epinephrine after recurrent hypoglycemia

**DOI:** 10.1371/journal.pone.0173839

**Published:** 2017-03-08

**Authors:** Juhye Lena Kim, Edmund F. La Gamma, Todd Estabrook, Necla Kudrick, Bistra B. Nankova

The image for Fig 6 is incorrect. The image should be in color. Please see the complete, correct Fig 6 here.

**Fig 6:**
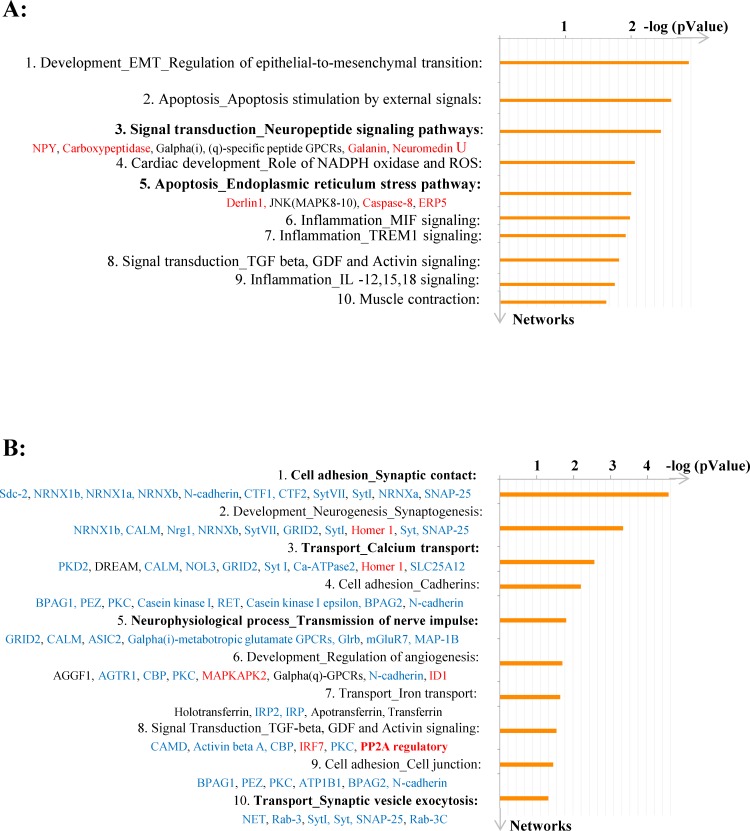
Enrichment analysis of DEGs in 2RH and 2RS experimental groups. A) Shown is the distribution by process networks at 0 time point (2RH0 vs. 2RS0) and B) at 60 min time point (2RH60 vs. 2RS60). Sorting is done for unique genes and both signals (induced genes—shown in red and repressed genes—in blue) included. Top 10 process networks are listed based on their—log (p-value). For list of abbreviations see supplemental file S1 Text.
