# Supplementary material for: Whole genome expression profiling associates activation of unfolded protein response with impaired production and release of epinephrine after recurrent hypoglycemia
Source: PLoS One. 2017 Feb 24;12(2):e0172789. doi: 10.1371/journal.pone.0172789 (PMC5325535; doi:10.1371/journal.pone.0172789)
Supplement: S1 Text — (DOCX) [file pone.0172789.s003.docx]

**Abbreviations for Figs 6 and 7:**

ERP5 (PDIA6) - protein disulfide isomerase family A member 6

Sdc-2 - syndecan 2

NRNX – neurexin

SYTVII - synaptotagmin VII

SYTI - synaptotagmin I

SNAP-25 - synaptosomal-associated protein 25 kDa

CALM – calmodulin

NRG1- neuregulin1

GRID2- glutamate receptor, ionotropic, delta 2

SLC25A12 - solute carrier family 25 (aspartate/glutamate carrier) member 12

PKD2- polycystic kidney disease 2

DREAM (KCNIP3) – potassium voltage-gated channel interacting protein 3

NOL3 (ARC) - nucleolar protein 3 (activity regulated cytoskeleton associated protein)

BPAG1 (DST) – dystonin

PKC - protein kinase C

RET- ret proto-oncogene

BPAG2 (COL17A1) - collagen type 17 alpha1

ASIC- acid-sensing ion channel

Glrb- glycine receptor, beta

mGluR7- metabotropic glutamate receptor 7

MAP-1B - microtubule-associated protein 1B

AGGF1- angiogenic factor with G patch and FHA domains 1

AGTR- angiotensin receptor

CBP- CREB-binding protein

MAPKAPK2 - mitogen-activated protein kinase-activated protein kinase 2

ID1- inhibitor of DNA binding 1

IRP - iron-responsive element binding proteins

IRF7 - interferon regulatory factor 7

NET - norepinephrine transporter

PP2A (PPP2R2B) – protein phosphatase 2, regulatory subunit B, beta

Rab3C – member, RAS oncogene family.

BAG-3- bcl2-associated athanogene 3

HSF1- heat shock transcription factor 1

DNAJB2- DnaJ (Hsp40) homolog, subfamily B, member 2

HSP22 (HSPB8) – heat shock 22kDa protein 8

HSPA1A- heat shock 70kDa Protein 1A

BAG4 (SODD) – silencer of death domains

HSPA1B - heat shock 70kDa protein 1B

TCP1-epsilon (CCT5) – chaperonin containing TCP1, subunit 5 (epsilon)

GADD45 gamma – growth arrest and DNA-damage inducible, gamma

TOPBP1 – topoisomerase 2 (DNA) binding protein 1

NFkB1 – nuclear factor of kappa light polypeptide gene enhancer in B-cells 1

BTG2 – B cell translocation gene 2

ATF3 – activating transcription factor 3

Bid – BH3 interacting domain death agonist

PPARd- peroxisome proliferator activated receptor delta

C/EBPb – CCAAT enhancer binding protein beta

SIAH2 – siah E3 ubiquitin protein ligase 2

IRS2 – insulin receptor substrate 2

EGR1- early growth response 1

SMAD3 - similar to the gene products of the Drosophila gene 'mothers against decapentaplegic' family member 3

WNT – wingless type MMTV integration site family member

Giot1- gonadotropin inducible transcription repressor 1

BTG1 – B-cell translocation gene 1, anti-proliferative

CREM – cAMP responsive element modulator

GADD34 (PPP1R15A) - protein phosphatase 1, regulatory subunit 15A

OASIS (CREB3L1) – cAMP responsive element binding protein 3-like 1

NUR77 (NR4A1)- nuclear receptor subfamily 4, group A, member1.
